# Novel Polyelectrolyte Complex Membranes Containing Carboxymethyl Cellulose–Gelatin for Pervaporation Dehydration of Azeotropic Bioethanol for Biofuel

**DOI:** 10.3390/polym14235114

**Published:** 2022-11-24

**Authors:** Prakash B. Kalahal, Ashok M. Sajjan, T. M. Yunus Khan, Ali A. Rajhi, Sharanappa Achappa, Nagaraj R. Banapurmath, Ashwini M, Alaauldeen A. Duhduh

**Affiliations:** 1 Department of Chemistry, KLE Technological University, Hubballi 580031, India; 2 Center for Material Science, KLE Technological University, Hubballi 580031, India; 3 Department of Mechanical Engineering, College of Engineering, King Khalid University, Abha 61421, Saudi Arabia; 4 Department of Biotechnology, KLE Technological University, Hubballi 580031, India; 5 AICRP on EAAI (Bioconversion Technology) MARS, University of Agricultural Sciences, Dharwad 580005, India; 6 Department of Mechanical Engineering Technology, CAIT, Jazan University, Prince Mohammed Street, Jazan 45142, Saudi Arabia

**Keywords:** pervaporation, flux, selectivity, permeance, permeability, activation energy

## Abstract

Polyelectrolyte complex membranes (PECMs) were prepared by combining sodium carboxymethyl cellulose (NaCMC) and gelatin (Ge) with variations in the Ge content in the NaCMC matrix. Characterization methods, such as infrared spectroscopy (FTIR), wide-angle X-ray diffraction (WAXD), thermogravimetric analysis (TGA), scanning electron microscopy (SEM), contact angle analysis (CA), and universal testing machines (UTM) were used to investigate the physicochemical studies of the prepared membranes. The pervaporation characteristics of membranes with Ge content were investigated using an azeotropic mixture of water and bioethanol. The obtained data revealed that the membrane with 15 mass% of Ge (M-3) showed a maximum flux of 7.8403 × 10^−2^ kg/m^2^·h with separation selectivity of 2917 at 30 °C. In particular, the total and water flux of PECMs are shown as very close to each other indicating that the fabricated membranes could be employed to successfully break the azeotropic point of water–bioethanol mixtures. Using temperature-dependent permeation and diffusion data, the Arrhenius activation parameters were calculated, and the obtained values of water permeation (E_pw_) were considerably smaller than bioethanol permeation (E_pE_). Developed membranes showed the positive heat of sorption (ΔH_s_), suggesting that Henry’s sorption mode is predominant.

## 1. Introduction

Renewable fuels have been gaining significance due to the depleting stocks of natural fuels in recent years [1]. In line with reproducible and green energy, bioethanol has greater energy efficiency, potentially substituting fossil fuels [2]. Specifically, bioethanol has a massive ability as a green renewable energy source due to its environmental aids and high efficacy [3]. Bioethanol is formerly dehydrated by the distillation route. However, excessive overheads, low efficiency, and higher energy consumption render the distillation process incompatible for the concentration of bioethanol [4]. Consequently, feasible, competent, and commercial bioethanol production has gained importance. In this context, bioethanol purification by membranes has gained importance due to its economical, convenient, and practical alternative [5].

Among the separation techniques, pervaporation (PV) is quite an effective method for the dehydration of organic substances, especially for separating azeotropic mixtures [6,7]. It has numerous benefits over the traditional distillation process, such as low cost, low energy intake, eco-friendly, and simplest operation [8,9].

PV dehydration efficiency depends mainly on the structure and properties of membranes [10]. According to the solution-diffusion model, the most important factor determining water’s selective sorption is the specific interaction between water and polymer. To obtain the high value of water permeation and selectivity, it is necessary to use polymers with high sorptive centers capable of specific interactions with water [11,12,13]. Some of the polymers, such as poly (acrylic acid) [14,15], poly (vinyl alcohol) [16,17], sodium alginate [18], hydroxyethyl cellulose [19], chitosan [20,21], and NaCMC have been recently applied for the fabrication of PV membranes. Among these membrane materials, NaCMC is a highly significant cellulose derivative; it is safe, non-toxic, biocompatible, and biodegradable. It has been used extensively in many industries, such as the paper, textile, food, cosmetics, chemical, and pharmaceutical sectors [22,23,24,25,26,27,28]. Along with these applications, it has outstanding film-forming capabilities, mechanical strength, chemical resistance, and high water selectivity [29]. However, membranes developed using this type of material posed a lack of mechanical strength and chemical stability in aqueous solutions mainly due to excessive swelling. Researchers focus on developing proficient PV membranes by identifying different membrane materials with exceptional properties. As a result, polyelectrolyte complex membranes (PECMs), which can assist PV separation with high flux and selectivity, have recently received special interest. PECMs, created by the ionic complexation of polyelectrolytes with opposing charges, are ideal membranes for organic dehydration [30,31,32,33].

Among the numerous membrane modifiers, gelatin (Ge) has gained attention due to its unique property. It is a natural protein derived from partially hydrolyzed collagen, a polyampholyte (free amino groups and carboxyl groups on its backbone) [34,35].

With this consideration in mind, Wang et al. [36] fabricated polyelectrolyte complex membranes using sulfated sodium carboxy methylcellulose with poly (diallyl dimethyl ammonium chloride) treated on polysulfone ultra-filtration membrane and observed a flux of 1.76 kg/m^2^ h, along with 98.7 wt% of water concentration in the permeate for SPECM-0.34 in dehydration of 10 wt% water–ethanol mixture at 70 °C. Zhao et al. [37] prepared soluble polyelectrolyte complexes between chitosan (CS) and sodium carboxymethyl cellulose membranes coated on polysulfone ultra-filtration membranes. HPECM-0.25 membrane exhibited a flux of 1.14 kg/m^2^ h, with a selectivity of 1062 for dehydrating 10 wt% water–ethanol mixture at 70 °C. Wang et al. [38] fabricated novel polyelectrolyte complexes containing chitosan/sodium carboxymethyl cellulose with polysulfone ultra-filtration membrane and studied for pervaporation separation of water–ethanol mixtures and PECSM-20 membrane attained higher flux of 1.38 kg/m^2^ h, with a separation factor of 1571 for dehydrating 10 wt% water–ethanol mixture at 70 °C. Among these membranes, polysulfone ultra-filtration membranes were used as supporting material. However, the use of Ge, which highlights the synergetic effect in the NaCMC membrane for dehydration of ethanol, has not been investigated. Therefore, in the current research, an effort has been made to develop novel polyelectrolyte complex membranes by incorporating various Ge contents in the NaCMC matrix. The physicochemical studies of the fabricated membranes were examined by various characterization methods, such as FTIR, WAXD, TGA, DSC, SEM, and UTM. The membranes were successfully employed for PV separation of the azeotropic bioethanol mixture. The values of permeation flux and separation selectivity were determined. Moreover, the diffusion coefficient and Arrhenius activation parameters were estimated. The results were discussed in terms of the PV separation efficiency of membranes.

## 2. Materials and Methods

### 2.1. Materials

Sodium carboxymethyl cellulose (NaCMC) (M.W~ 90,000) was obtained from Sigma- Aldrich St. Louis, MO, USA. Gelatin (Ge) was procured from S.D. fine chemicals Ltd., Mumbai, India. Rectified bioethanol was obtained from Godavari Biorefineries, Karnataka, India. Hydrochloric acid was purchased from E. Merck Ltd., Mumbai, India. All the chemicals were of reagent grade and were utilized directly. Double distilled water was used for the entire investigation.

### 2.2. Fabrication of PECMs

**Figure 1:**
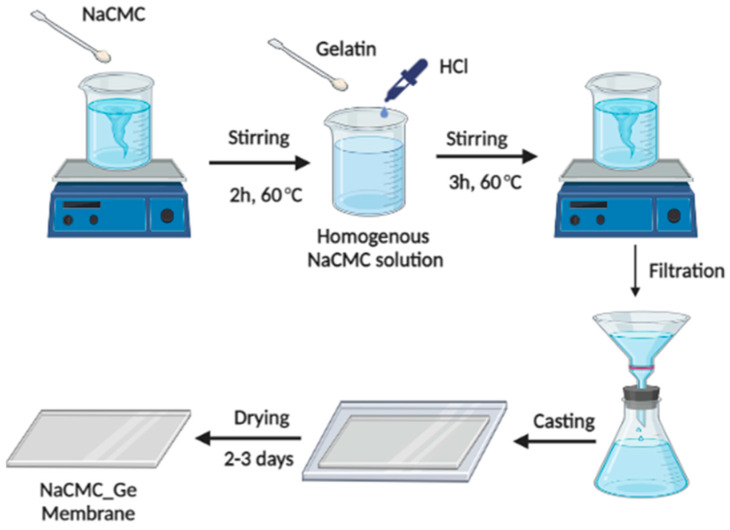
Scheme of the preparation of Ge-incorporated NaCMC membranes.

NaCMC–Ge PECMs were developed by the solution casting method. Herein, 3 wt% of NaCMC was added to 100 mL of double distilled water by stirring for 2 h at 60 °C. The subsequent homogeneous solution was filtered and spread onto a glass plate. After drying for 2–3 days at ambient temperature, the membrane was peeled-off from the glass plate and labeled as M. A known mass% (5, 10, 15, and 20 mass%) of Ge and concentrated HCl (1 mL) were added to the homogeneous NaCMC solution and it was maintained in an oil bath for 3 h at 60 °C. The rest of the process was followed in similar way to membrane M. The obtained membranes were M-1, M-2, M-3, and M-4, respectively. The thickness of the fabricated PECMs was evaluated by a Peacock dial thickness gauge, and it was observed to be 50 ± 2 µm. The preparation scheme of the Ge-incorporated NaCMC membrane is represented in Figure 1.

### 2.3. Characterization of PECMs

The physicochemical studies of the fabricated membranes were examined by various characterization methods, such as Fourier transform infrared (FTIR) spectroscopy, wide-angle X-ray diffraction (WAXD), thermogravimetric analysis (TGA), differential scanning calorimetry (DSC), scanning electron microscopy (SEM), universal testing machine (UTM), and contact angle (details of characterization techniques can be found in supporting information).

### 2.4. Degree of Swelling (DS)

Developed membranes were weighed after drying in an electronically controlled oven (Tempo instruments, Mumbai, India). Furthermore, membranes were placed in a closed bottle containing an azeotropic mixture for 24 h at room temperature. After attaining equilibrium, membranes were taken out, blotted, and weighed. The following expression % sorption of membranes was calculated as [39,40,41]:

(1) $$DS\;(\%)=\left(\frac{W_{s}-W_{d}}{W_{d}}\right)\;\times \;100$$

where *W_d_* and *W_s_* are the masses of the dry and swollen membranes, respectively.

### 2.5. Pervaporation Experiments

Indigenously designed apparatus for PV experiments was used by following the procedure reported in our previous studies [42]. The developed membrane, with a surface area of 34.21 cm^2^ and a thickness of 50 µm, was used for the PV experiments. The experiments were conducted in triplicate by maintaining a vacuum pressure of 1.333224 × 10^3^ Pa (10 Torr) downstream of the PV cell with a dual-stage vacuum pump (Toshniwal, Chennai, India). The PV performance of the developed PECMs was assessed by determining the permeation flux (J), separation selectivity (αsep), total pervaporation separation index (PSI), and permeance (Pi/l) [43,44,45,46].

## 3. Results

### 3.1. Characterization of PECMs

#### 3.1.1. FTIR Analysis

**Figure 2:**
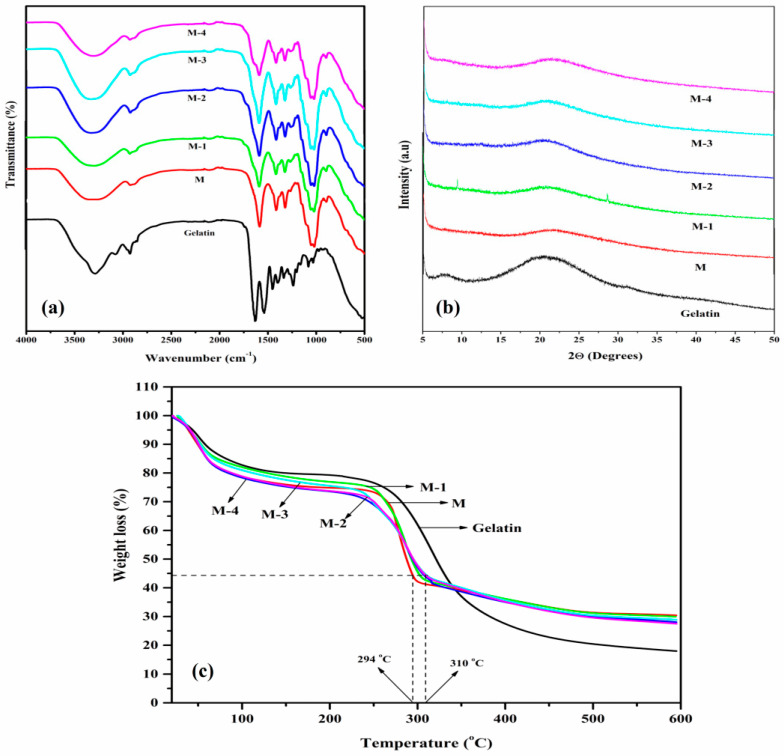
(**a**) FTIR spectra; (**b**) wide-angle X-ray diffraction; (**c**) thermogravimetric analysis patterns of Ge; NaCMC (M); and Ge-incorporated NaCMC membranes: (M-1) 5 mass%; (M-2) 10 mass%; (M-3) 15 mass%; (M-4) 20 mass% of Ge.

Figure 2a represents the FTIR spectra of pure Ge, NaCMC, and Ge-incorporated NaCMC membranes. In the plain NaCMC membrane, the broadband appeared at 3318 cm^−1^ due to O-H stretching, the band at 2910 cm^−1^ represents aliphatic C-H stretching, and the bands at 1585 and 1411 cm^−1^ were due to the symmetric and asymmetric stretching of carboxylate groups, respectively. The absorption bands at 1322 and 1050 cm^−1^ indicate the O-H bending vibrations and C-O-C bond stretching vibrations [47]. In the plain Ge membrane, the broadband appeared at 3295 cm^−1^ due to NH_2_ stretching, and the band observed at 1636 cm^−1^ signifies C=O stretching of the amide band. Moreover, the presence of a characteristic band at 1537 cm^−1^ indicates N-H bending vibrations, and the absorption band at 1238 cm^−1^ represents C-N stretching vibrations [48].

**Figure 3:**
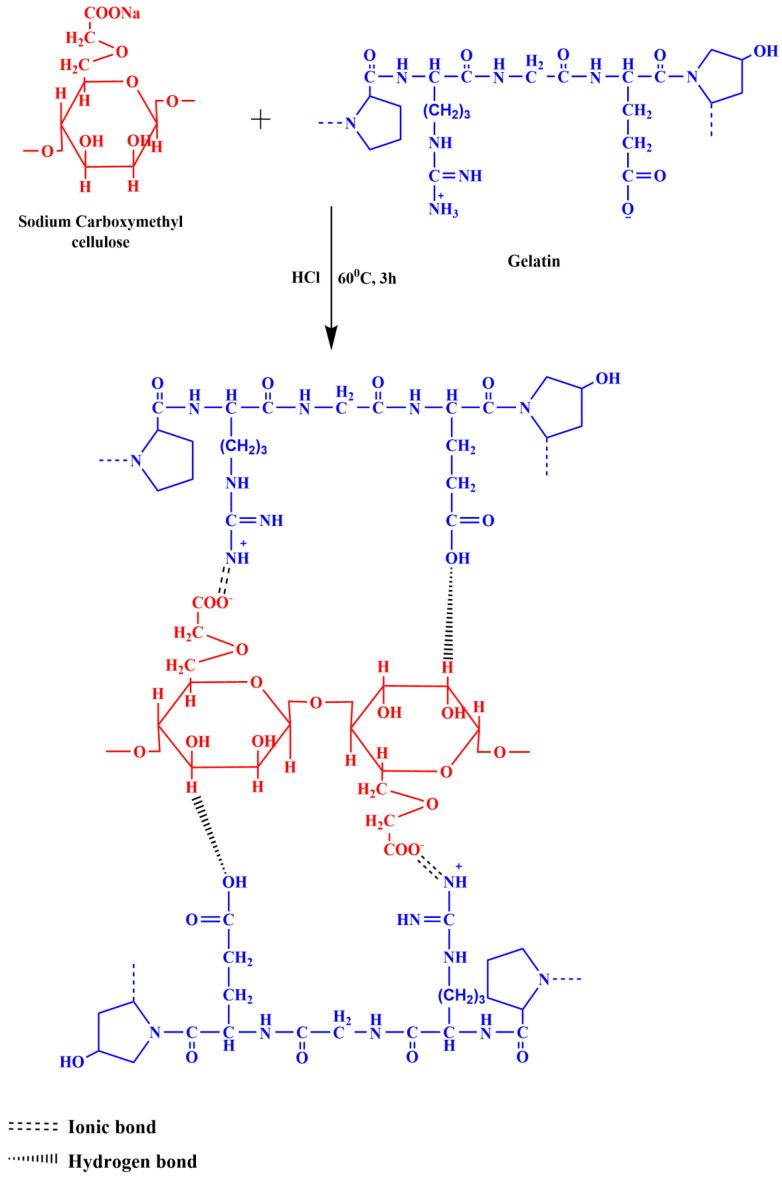
Interaction between NaCMC and Ge.

In the Ge-incorporated NaCMC membranes, the band assigned to O-H stretching was broadened, suggesting their involvement in forming intermolecular hydrogen bonding with Ge. Additionally, it was found that the intensity of the strong band that appeared at 1585 cm^−1^ of NaCMC membrane was reduced and broadened as Ge content increased. This is due to the electrostatic attraction between the -COO^−^ group of NaCMC and the -NH_3_^+^ group of Ge. The reactions between NaCMC and Ge are shown in Figure 3.

#### 3.1.2. WAXD Analysis

The diffractograms of plain Ge, NaCMC, and Ge-incorporated NaCMC membranes are shown in Figure 2b. There were two peaks observed in the gelatin at 7.71° and 20.60°. The small peak at 7.71° indicates the triple helix diameter of the Ge. The peak at 20.60° is due to the semi-crystalline character of the Ge [49,50]. In the NaCMC (M) membrane, the broad peak at about 21.43° is due to the low crystallinity of the NaCMC structure [51]. In Ge-incorporated NaCMC membranes, the peak 7.71° of Ge disappeared which indicates that the triple helix structure of Ge is disturbed. Moreover, the peak of 21.43° of NaCMC was shifted to a lower degree (20.78°), and the peak intensity was augmented up to 15 mass% of Ge. For 20 mass% of Ge (M-4), the peak was observed at 21.50° with a decrease in the peak’s intensity. This is due to the fact that the incorporated Ge is aggregated with each other, leading to the entanglement with the NaCMC chain through hydrogen bonding and electrostatic interaction between -COO^−^ and -NH_3_^+^ of NaCMC and Ge. Furthermore, the d-spacing values corresponding to the 21.43° of NaCMC and Ge-incorporated NaCMC membranes were 4.14, 4.27, 4.29, 4.26, and 4.13 Å. The increase in crystallinity degree and d-spacing results from systematic polymer chain packing with free space between polymer chains. Higher crystallinity degree and d-spacing imply increased chain packing and free space between the polymer chains, thereby increasing the selectivity and permeability of the component.

#### 3.1.3. TGA Analysis

The thermograms of the Ge, NaCMC, and Ge-incorporated NaCMC membranes are shown in Figure 2c. All of the analyses were performed under the nitrogen flow. The thermal decomposition of the Ge-incorporated NaCMC membranes was carried out using three steps. In the initial step, the weight loss ensued between ambient temperatures to 200 °C due to the absorbed water molecules. The majority of these absorbed water molecules were observed to be directly linked to the polymer chains and not in a free molecular state. This weight loss is about 25% for NaCMC membrane, whereas membranes containing 5 mass% (M-1) of Ge exhibited a lower loss of about 23%. This signifies that the incorporated Ge is involved in ionic-cross linking between the ionic groups, such as -COO^−^ and -NH_3_^+^ of NaCMC and Ge, and clearly, the resulting membrane exhibited lower weight loss than the NaCMC membrane.

On the other hand, membranes incorporated with 15 mass% of Ge exhibited higher weight loss (27%). This is due to the fact that the incorporated Ge is not completely involved in ionic-cross linking and may be due to repulsion between the -NH_3_^+^ groups of Ge, which creates the free binding sites in the membrane matrix for water molecules. However, membranes incorporated with 20 mass% of Ge showed higher weight loss (29%). This is due to the fact that the incorporated Ge is aggregated with each other, leading to the entanglement with the NaCMC chain through hydrogen bonding and electrostatic interaction between -COO^−^ and -NH_3_^+^ of NaCMC and Ge. In the second step, the decomposition occurred between the temperature range of 230 to 310 °C due to the decomposition of the functional groups of NaCMC and Ge. Subsequently, the breaking of the polymer chain caused the third stage of decomposition, which was visible between the temperature range of 310 to 520 °C. By considering 56% degradation as the reference point, the plain NaCMC membrane showed degradation at the temperature of 294 °C and the M-3 membrane at 310 °C. This indicates the highest interaction between NaCMC and Ge in M-3, which leads to the highest thermal stability. However, from the thermogram, it is clear that the Ge-incorporated NaCMC membranes are more stable than the pure NaCMC membranes due to the intermolecular interaction between NaCMC and Ge.

#### 3.1.4. SEM Analysis

**Figure 4:**
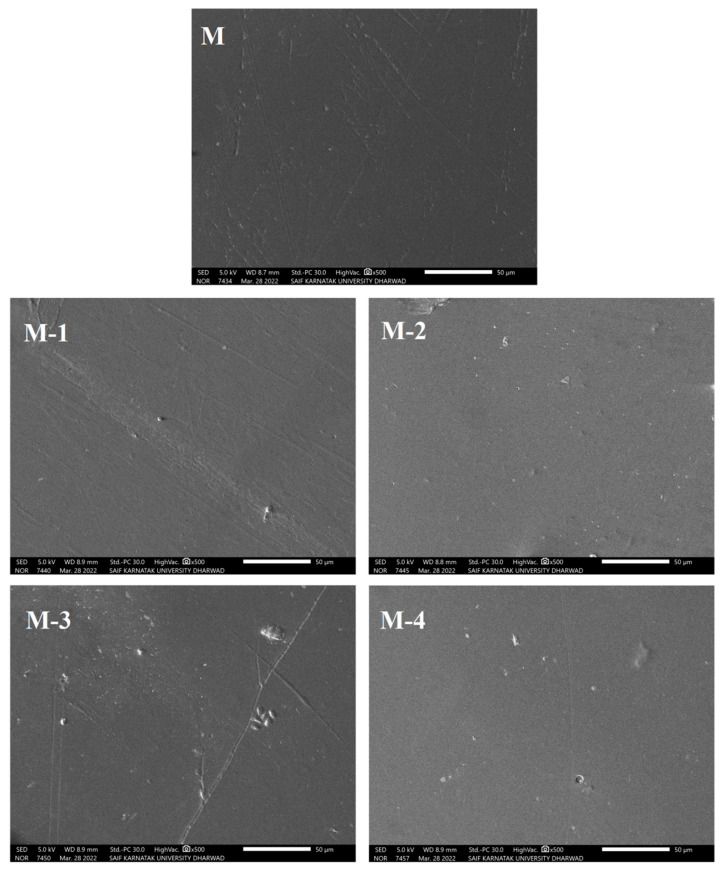
SEM micrographs of NaCMC and its Ge-incorporated PECM membranes; (**M**) 0 mass%; (**M-1**) 5 mass%; (**M-2**) 10 mass%; (**M-3**) 15 mass%; (**M-4**) 20 mass% of Ge.

The surface morphology of the developed PECMs was assessed by SEM analysis, and the subsequent photographs are shown in Figure 4.

It is observed that membranes are smooth and homogeneous with no porosity, and there is no sign of phase separation in the Ge-incorporated NaCMC membrane. This indicates a complete interaction between NaCMC and Ge and shows good compatibility between the two moieties.

#### 3.1.5. Tensile Strength

The mechanical characteristics are crucial in evaluating the steadiness of the developed membranes for PV applications. Elongation at break and tensile strength values of the developed PECMs are shown in Table 1.

Due to the extensive ionic interaction and hydrogen bonding between the NaCMC and Ge, the mechanical properties of developed membranes are greatly enhanced as the Ge content increases. As the concentration of Ge is enhanced, a significant increase in the tensile strength from 28.640 to 31.925 MPa was shown, along with maintaining decent elongation at break. This clearly shows that the mechanical strength of the membrane is influenced by the interaction of the NaCMC matrix with Ge. It makes the membrane denser, which results in increased tensile strength. All the membranes have shown higher mechanical strengths and are highly capable of handling the applied vacuum in the pervaporation experiments.

#### 3.1.6. Contact Angle Analysis

**Figure 5:**
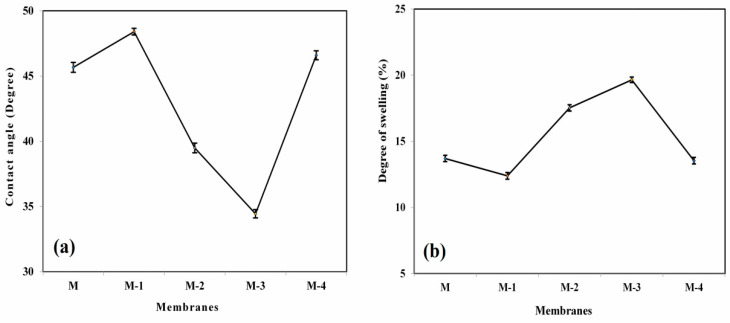
(**a**) Contact angle; (**b**) degree of swelling values of NaCMC, and its Ge-incorporated PECM membranes; (M) 0 mass%; (M-1) 5 mass%; (M-2) 10 mass%; (M-3) 15 mass%; (M-4) 20 mass% of Ge.

Contact angle values show a significant part in evaluating the hydrophilic nature, which influences the permeation flux of the developed membrane. Figure 5a illustrates the consequence of the Ge effect with the contact angle values of the developed PECM membranes. The contact angle value of 45.81° was observed for the plain NaCMC membrane (M). After incorporating Ge in the NaCMC matrix, the contact angle value was increased for M-1. This is due to the compactness of polymer chains, which leads to ionic cross-linking and inter- and intra-molecular hydrogen bonding between the ionic groups, such as -COO^−^ and NH_3_^+^ of NaCMC and Ge. However, the contact angle was decreased when adding Ge up to 15 mass% (M-3). This is due to the dominant nature of the repulsion between the -NH_3_^+^ group of Ge, which leads to higher water affinity. However, a further increase in Ge content beyond 15 mass% (M-4) significantly increased the contact angle value, leading to a reduction in the free volume and hydrophilic character of PECMs, due to the ionic interaction between the -COO^−^ and NH_3_^+^ groups of NaCMC and Ge.

### 3.2. Influence of Ge Content on Membrane Swelling

In order to study the effect of Ge content on membrane swelling, the sorption experiment was carried out, and the calculated values are plotted with respect to mass % of Ge (Figure 5b). The degree of swelling for pure NaCMC membrane was 13.94%. The initial addition of 5 mass% of Ge decreased the DS. This is due to the fact that the added Ge contains -NH_3_^+^, which establishes the ionic cross-link with -COO^−^ groups of the NaCMC matrix, leading to a more compact structure and making the free carboxyl groups of NaCMC unavailable for solvent interaction [52,53]. However, when the content of Ge was increased to 15 mass%, the degree of swelling was increased, which is due to the repulsion that exists between the -NH_3_^+^ group of Ge. Therefore, the network structure of membrane M-3 has more potential for hydrogen bonding with the surrounding water.

Further increasing the Ge to 20 mass% decreases the degree of swelling. This may be due to the ionic interaction established between the -COO^−^ and NH_3_^+^ groups of NaCMC and Ge, respectively. The degree of swelling results is in line with WAXD and TGA data.

### 3.3. Inflence of Ge Content on PV

**Figure 6:**
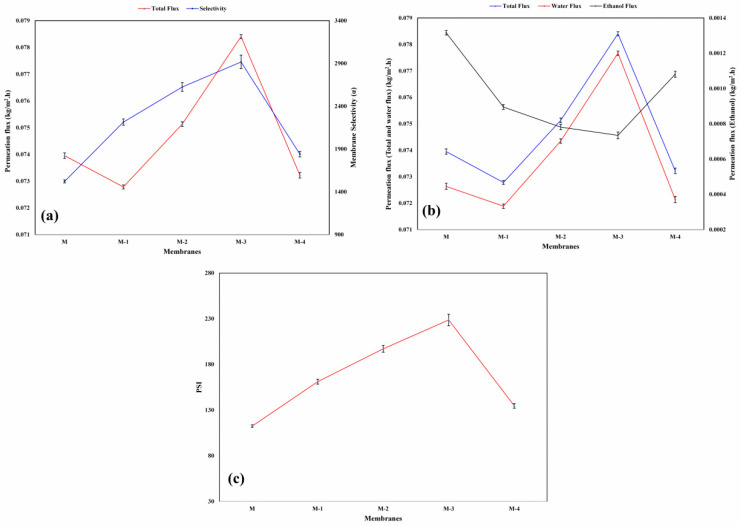
(**a**) Deviation in total permeation flux and separation selectivity with different mass% of Ge; (**b**) deviation in total permeation flux and permeation fluxes of water and ethanol at 30 °C; (**c**) effect of Ge content on pervaporation separation index (PSI) at 30 °C.

Figure 6a illustrates the effects of Ge content on the total permeation flux and separation selectivity of water and ethanol. The observed values of permeation flux decreased initially with the addition of Ge of 5 mass% (M-1). This is due to the fact that -NH_3_^+^ of Ge establishes the ionic cross-link with -COO^−^ groups of the NaCMC matrix, leading to a more compact structure and making the free carboxyl groups of NaCMC unavailable for solvent interaction. The permeation flux was enhanced by adding Ge up to 15 mass% (M-3), which is due to a repulsive force existing between the -NH_3_^+^ group of Ge, thereby forming a potential hydrogen bond between the surrounding water leading to higher water affinity and permeation flux. With a further increase in Ge content to 20 mass% (M-4), the permeation flux decreased due to the ionic interaction between Ge and NaCMC, thereby lowering the hydrophilic nature of the membrane. This is due to the fact that the incorporated Ge is aggregated with each other, leading to the entanglement with the NaCMC chain through hydrogen bonding and electrostatic interaction between -COO^−^ and -NH_3_^+^ of NaCMC and Ge. As observed, the change in permeation flux is in agreement with the WAXD, TGA, and degree of swelling results.

The separation selectivity of the membrane largely depends on the interaction between the membrane and the permeating species, the molecular size of the permeating species, and the pore size of the membrane. Figure 6a illustrates the effect of Ge content in the NaCMC matrix on separation selectivity. The separation selectivity of the membranes was systematically increased with the addition of the Ge up to 15 mass%. This is due to the ionic cross-linking and inter- and intra-molecular hydrogen bonding between the ionic groups, such as -COO^−^ and -NH_3_^+^ of NaCMC and Ge, respectively, leading to the high water selective membranes. With a further increase in Ge content to 20 mass%, the membrane selectivity substantially declined due to the lower hydrophilic behavior. Additionally, the incorporated Ge is aggregated with each other, leading to the formation of defects and the rigidity of NaCMC chains increases.

Figure 6b illustrates the effect of Ge content on the total permeation flux and permeation fluxes of water and ethanol. The curves of the total permeation flux and water permeation flux are close to each other, indicating the hydrophilic behavior of the fabricated membrane. The permeation curve of ethanol is comparatively very low, thereby indicating that the membrane is selective toward the water. The trade-off phenomenon of the developed membrane was not observed, as indicated by augmentation in separation selectivity and permeation flux (from M-1 to M-3) with an increase in the Ge content in the membrane matrix.

The permeance and permeability of water and bioethanol are represented in Table 2 and Table 3, respectively. The values of permeance and permeability of water and bioethanol are in line with the data of permeation flux and degree of swelling. The permeance and permeability of water decreased initially with the addition of Ge (M-1). This is due to the compactness of polymer chains, as a result of ionic cross-linking and inter- and intra-molecular hydrogen bonding between the ionic groups, such as -COO^−^ and -NH_3_^+^ of NaCMC and Ge. With a further increase in Ge up to 15 mass%, the permeance and permeability increased. This is due to the dominant nature of the repulsion that exists between the -NH_3_^+^ group of Ge, which leads to higher permeance and permeability. However, with an increase in the Ge content above 15 mass%, the permeance and permeability decreased due to the lower hydrophilic nature and reduced free volume.

### 3.4. Influence of Ge Content on PSI

To evaluate the separation efficiency of the fabricated membranes, PSI was used, and is governed by permeation flux and separation selectivity. The graph of PSI values at different contents of Ge at 30 °C is shown in Figure 6c. According to the graph, the PSI values enhanced steadily due to the higher permeation flux and separation selectivity as the content of Ge increased up to 15 mass% (M-3) in the membrane. However, adding Ge beyond 15 mass% significantly decreased the selectivity and flux due to the decline in the hydrophilic nature that leads to the decrease in the PSI value of membrane M-4. Membrane M-3 revealed the maximum PSI value (229) of the developed membranes, which implies that it shows excellent PV performance.

### 3.5. Comparison of PV Performance with the Literature

Literature review of flux and selectivity data of different polymeric membranes for dehydration of water–bioethanol mixture are summarized in Table 4. It is noticed that membranes created by Na-Alg/3.0% Ag_Nps-PSSAMA_Na, PVA/PSStSA-co-MA, PVDF/chitosan-alginate, ECN silica membrane, Nafion-H^+^, Nafion-Na^+^, Nafion-K^+^, PAN–PVP, cationic PVA/GA, anionic PVA/GA, PVA/GA, PVA/GA acrylic acid, unmodified PVA, cellulose acetate, and Teflon-g-PVP exhibited higher permeation flux compared to Ge-incorporated NaCMC membranes, but their selectivity was significantly lower (3-1140). On the other hand, membranes created by chitosan/PAA, chitosan, and chitosan acetate salt exhibited good separation selectivity with the least permeation flux. The membranes created by polystyrene, PVC, and chitosan/GA exhibited very low separation selectivity compared to the present work. The fabricated membranes showed excellent permeation flux and separation selectivity at ambient temperature due to the strong ionic interaction, leading to the formation of a polymer electrolyte complex between NaCMC and Ge.

### 3.6. Diffusion Coefficient

Determining the diffusion coefficients of penetrating molecules is crucial for understanding the molecular transport mechanism. The diffusion flux is defined by Fick’s equation of diffusion [69]:

(2) $$J_{i}=-D_{i}\frac{dC_{i}}{dx}$$

where *J* is the flux of *i*th component (kg/m^2^ s), *D* is the diffusion coefficient (m^2^/s), *C* is the concentration of permeant (kg/m^3^), subscript *i* stands for water or ethanol, and *x* is the diffusion length (m). For simplicity, it is assumed that the concentration profile along the diffusion length is linear. Therefore, *D_i_* can be calculated with the following equation [70]:

(3) $$D_{i}=\frac{J_{i}\delta }{C_{i}}$$

where δ represents the thickness of the membrane.

In Table 5, the water diffusion coefficient value is high compared to the diffusion coefficient of ethanol for all the membranes. For 5 mass% Ge (M-1), the water diffusion coefficient was decreased, which is due to the compactness of polymer chains, leading to the ionic cross-linking and inter- and intra-molecular hydrogen bonding between the ionic groups, such as -COO^−^ and -NH_3_^+^ of NaCMC and Ge. On further addition of Ge up to 15 mass%, the water diffusion coefficient increased. This is due to the dominant nature of the repulsion that exists between the -NH_3_^+^ group of Ge, leading to a higher water diffusion coefficient. However, increasing the Ge content above 15 mass% will reduce the diffusion coefficient of water due to the lower hydrophilic nature and free volume.

### 3.7. Effect of Temperature on Membrane Performance

The impact of temperature on PV performance for water–bioethanol mixtures was investigated for all membranes, and the obtained results are shown in Table 6. A substantial increase in overall penetration flux from 30 to 50 °C, while a reduction in the selectivity of all membranes leads to the estimation of the activation energy for permeation using the Arrhenius type equation [71]:

(4) $$X=X_{0}\exp\;\left(\frac{-E_{X}}{\mathrm{RT}}\right)$$

where X_0_ represents the pre-exponential factor, E_X_ represents the activation energy for permeation or diffusion, R is the gas constant, and T is the temperature.

Two-way ANOVA with replication was performed at a 95% (α = 0.05) confidence level to observe the effect of membrane type and temperature on permeation flux and selectivity, as represented in Table 7. Based on the *p*-value for membrane type (*p* = 5.3 × 10^−25^ and 5.99 × 10^−32^ < 0.05 = α) and temperature (*p* = 6.72 × 10^−17^ and 2.76 × 10^−34^ < 0.05 = α), an interaction effect between membrane type and temperature (*p* = 0.0113 and 1.34 × 10^−21^ < 0.05 = α) shows that the factors are statistically significant by rejecting the null hypothesis. To observe the difference in means of the parameters between membrane type and temperature, the post-hoc test using Tukey’s HSD was performed after two-way ANOVA. Tukey’s test on membrane type showed a significant effect on total permeation flux and selectivity, as the means between all membranes are higher than the mean critical of 0.000559 for total permeation flux and 45.54 for selectivity, respectively. Similarly, temperature showed a significant effect on total permeation flux and selectivity, as the means between all temperatures are higher than the mean critical of 0.000368 for total permeation flux and 29.9817 for selectivity, respectively. However, Tukey’s test showed no significant effect between membrane type and temperature for total permeation flux and selectivity.

**Figure 7:**
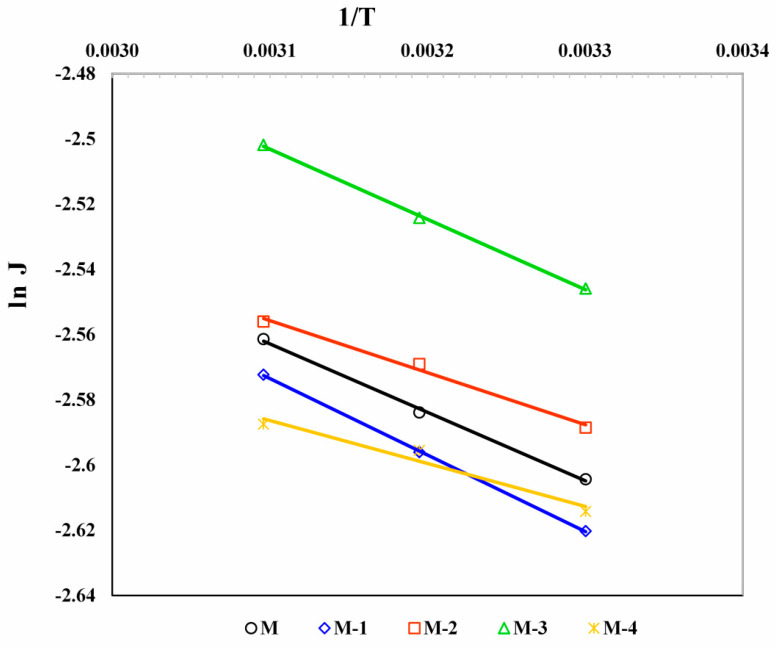
Effect of Ge content in NaCMC membranes on flux with temperature.

**Figure 8:**
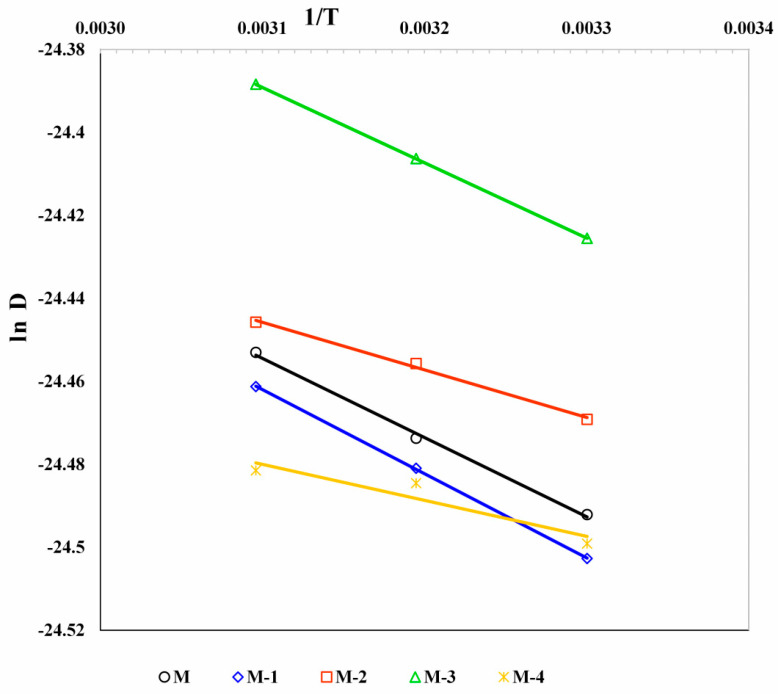
Effect of Ge content in NaCMC membranes on diffusivity with temperature.

The Arrhenius plots for the temperature dependence of permeation flux and diffusion are displayed in Figure 7 and Figure 8. The activation energies for total permeation (E_p_) and diffusion (E_D_) were derived from the least-squares fits of these linear plots. Similar activation energies were estimated for the diffusion of water (E_Dw_) and bioethanol (E_DET_), as well as the permeation of water (E_pw_) and bioethanol (E_pET_). However, the plots were not included to minimize crowding. The obtained values are shown in Table 8.

According to Table 8, the E_p_ and E_pw_ values are close to each other. However, there was a significant difference between the E_pw_ and E_pET_, suggesting that the united transport of both (water and bioethanol) is negligible due to the maximum separation efficiency toward the water. For all membranes, E_p_ and E_D_ values are shown as insignificant differences, which signifies that sorption and diffusion play an equal role in the PV experiment.

The E_p_ and E_D_ values ranged between 1.33 and 1.95, and 0.72 and 1.69 kJ/mol, respectively. Using these values, the heat of sorption is calculated as [72]:

(5) $$\Delta H_{s}=E_{p}-E_{D}$$

The obtained ∆H_s_ values are shown in Table 8. The conveyance of molecules across the polymer matrix was assessed by ∆H_s_ values [73]. All the membranes revealed positive ΔH_s_ values, which signifies that Henry’s mode of sorption is predominantly dominated by an endothermic impact.

## 4. Conclusions

Polyelectrolyte complex membranes (PECMs) were developed by combining NaCMC and Ge with variations in the Ge content in the NaCMC matrix. The membrane performance on separation of azeotropic bioethanol mixture was systematically evaluated at different temperatures. This study showed that an increase in the content of Ge up to 15 mass% in NaCMC matrix increases water transportation across the membrane with a total flux of 7.8403 × 10^−2^ kg/m^2^ h at 30 °C and selectivity of 2917 as well as through limiting the bioethanol permeation. Further increase in Ge content up to 20 mass% showed a decrease in the permeation of water. These data are followed by sorption studies and contact angle measurements. The experimental data of the total and water permeation flux are close to each other, indicating the hydrophilic behavior of the fabricated membrane. The permeation data of ethanol are comparatively very low as indicated, which signifies the selectivity of the membrane for water. The trade-off phenomenon of the developed membrane was not observed as indicated by augmentation in both separation selectivity and permeation flux (from M-1 to M-3) with an increase in the Ge content in the membrane matrix. The PECMs exhibited significantly lower water permeation (E_pw_) values than bioethanol permeation (E_pE_). The assessed E_p_ and E_D_ values ranged between 1.33 and 1.95, and 0.72 and 1.69 kJ/mol, respectively. The obtained positive heat of sorption (ΔH_s_) values suggest that Henry’s mode of sorption attributed to an endothermic contribution.

## Figures and Tables

**Table 1:** Mechanical properties of the Ge-incorporated NaCMC membranes.

Membrane	Tensile Strength (MPa)	Elongation at Break (%)
M	22.570 ± 2.60	3.745 ± 1.71
M-1	28.640 ± 3.18	3.322 ± 1.96
M-2	30.672 ± 3.27	4.021 ± 2.83
M-3	31.031 ± 2.19	4.529 ± 2.14
M-4	31.925 ± 2.40	4.695 ± 1.20

**Table 2:** The permeance of water and bioethanol of all the membranes at different temperatures.

**Temp. °C**		**P_w_/l (GPU)**			
	**M**	**M-1**	**M-2**	**M-3**	**M-4**
30	2017.63 ± 3.20	1996.85 ± 2.28	2065.23 ± 2.35	2157.46 ± 2.34	2003.86 ± 3.32
40	2054.88 ± 2.60	2040.59 ± 0.94	2092.53 ± 3.00	2199.01 ± 3.03	2032.82 ± 28.98
50	2097.63 ± 1.91	2080.73 ± 2.28	2113.18 ± 3.17	2238.27 ± 3.25	2038.54 ± 28.35
**Temp. °C**		**P_E_/l (GPU)**			
	**M**	**M-1**	**M-2**	**M-3**	**M-4**
30	46.28 ± 0.45	31.48 ± 0.49	27.47 ± 0.54	25.82 ± 0.68	38.02 ± 0.59
40	52.90 ± 0.37	38.80 ± 0.80	44.99 ± 0.51	33.52 ± 0.54	50.01 ± 0.27
50	59.08 ± 0.90	51.01 ± 0.50	54.13 ± 0.64	47.53 ± 0.51	64.00 ± 1.03

GPU = gas permeation unit = 10^−6^ cc (STP)/cm^2^/s/cm Hg.

**Table 3:** The permeability of water and bioethanol of all the membranes at different temperatures.

**Temp. °C**		**Pi_w_ (Barrer) (10^4^)**			
	**M**	**M-1**	**M-2**	**M-3**	**M-4**
30	8.07 ± 0.012	7.99 ± 0.009	8.26 ± 0.009	8.63 ± 0.009	8.02 ± 0.013
40	8.22 ± 0.010	8.16 ± 0.003	8.37 ± 0.012	8.37 ± 0.012	8.13 ± 0.115
50	8.39 ± 0.007	8.32 ± 0.009	8.45 ± 0.012	8.95 ± 0.013	8.15 ± 0.113
**Temp. °C**		**Pi_E_ (Barrer) (10^3^)**			
	**M**	**M-1**	**M-2**	**M-3**	**M-4**
30	1.85 ± 0.018	1.26 ± 0.019	1.10 ± 0.021	1.03 ± 0.027	1.52 ± 0.023
40	2.12 ± 0.015	1.55 ± 0.032	1.80 ± 0.020	1.34 ± 0.021	2.00 ± 0.011
50	2.36 ± 0.036	2.04 ± 0.020	2.17 ± 0.025	1.90 ± 0.020	2.56 ± 0.041

Barrer = 1 × 10^−10^ cc (STP)/cm^2^·s·cm Hg.

**Table 4:** Pervaporation performance of different membranes for dehydration of ethanol at azeotropic point ^a^.

Membranes	Temp. (°C)	Permeation Flux (J) (kg/m^2^h)	Separation Selectivity (α_sep_)	Ref.
Na-Alg/3.0% Ag_Nps-PSSAMA_Na	30	0.134	1140	[54]
PVA/PSStSA-co-MA	30	0.43	190	[55]
Chitosan/PAA	30	0.033	2216	[56]
PVDF/Chitosan-Alginate	50	0.095	202	[57]
ECN silica membrane	70	1.6	350	[58]
Nafion-H^+^	70	5	2.5	[59]
Nafion-Na^+^	70	0.5	5	[59]
Nafion-K^+^	70	0.2	9.8	[59]
PAN–PVP	20	2.2	3.2	[60]
Polystyrene	40	0.005	101	[61]
PVC	40	0.003	63	[62]
Alginic acid	40	0.048	8.8	[63]
Chitosan	40	0.004	2208	[64]
Chitosan acetate salt	40	0.002	2556	[65]
Chitosan/GA	40	0.007	202	[65]
Cationic PVA/GA	40	0.089	709	[66]
Anionic PVA/GA	40	0.086	837	[66]
PVA/GA	40	0.189	335	[66]
PVA/GA acrylic acid	40	0.135	14	[67]
Unmodified PVA	40	0.091	15	[67]
Cellulose acetate	60	0.2	5.9	[68]
Teflon-g-PVP	25	2.2	2.9	[68]
NaCMC (M)	30	0.073	1521	Present work
NaCMC/5 mass% Ge (M-1)	30	0.072	2214	Present work
NaCMC/10 mass% Ge (M-2)	30	0.075	2624	Present work
NaCMC/15 mass% Ge (M-3)	30	0.078	2917	Present work
NaCMC/20 mass% Ge (M-4)	30	0.073	1839	Present work

^a^ P-HPA: Preyssler heteropolyacid, Na-Alg: Sodium alginate, PSSAMA_Na: Poly (styrene-4-sulfonic acid co maleic acid sodium salt), Ag_Nps: Silver nanoparticles, GA: Glutaraldehyde, PVA: Poly (vinyl alcohol), PSStSA-co-MA: Poly (sodium salt styrene sulfonic acid-co-maleic acid), PAA: Polyacrylic acid, PVDF: Poly (vinylidene fluoride), NaCMC: Sodium carboxymethyl cellulose, Ge: Gelatin.

**Table 5:** The diffusion coefficients of water and bioethanol of all the membranes at different temperatures.

**Temp. °C**		**D_w_ (10^−11^ m^2^/s)**			
	**M**	**M-1**	**M-2**	**M-3**	**M-4**
30	2.31 ± 0.0036	2.28 ± 0.0026	2.36 ± 0.0026	2.47 ± 0.0026	2.29 ± 0.0037
40	2.35 ± 0.0029	2.33 ± 0.0010	2.39 ± 0.0034	2.51 ± 0.0034	2.32 ± 0.0033
50	2.40 ± 0.0021	2.38 ± 0.0026	2.42 ± 0.0036	2.56 ± 0.0037	2.33 ± 0.0032
**Temp. °C**		**D_E_ (10^−14^ m^2^/s)**			
	**M**	**M-1**	**M-2**	**M-3**	**M-4**
30	1.92 ± 0.018	1.30 ± 0.020	1.14 ± 0.022	1.07 ± 0.028	1.58 ± 0.024
40	2.19 ± 0.015	1.61 ± 0.033	1.86 ± 0.021	1.39 ± 0.022	2.07 ± 0.011
50	2.45 ± 0.037	2.11 ± 0.021	2.24 ± 0.026	1.97 ± 0.021	2.65 ± 0.043

**Table 6:** Pervaporation flux and separation selectivity of all the membranes at different temperatures.

**Membrane**	**Temperature (°C)**	**J × 10^−2^** **(kg/m^2^h)**	**α_sep._**	**P_i_/l** **(GPU)**
	30	7.3950 ± 0.00010	1521.52 ± 17.40	2017.63 ± 3.20
M	40	7.5480 ± 0.00008	1355.66 ± 10.57	2054.88 ± 2.60
	50	7.7195 ± 0.00009	1239.28 ± 17.70	2097.63 ± 1.91
	30	7.2781 ± 0.00008	2214.40 ± 36.45	1996.85 ± 2.28
M-1	40	7.4564 ± 0.00003	1835.87 ± 37.78	2040.59 ± 0.94
	50	7.6357 ± 0.00008	1423.66 ± 15.27	2080.73 ± 2.28
	30	7.5129 ± 0.00008	2624.18 ± 51.00	2065.23 ± 2.35
M-2	40	7.6610 ± 0.00010	1623.57 ± 19.77	2092.53 ± 3.00
	50	7.7613 ± 0.00011	1362.73 ± 17.59	2113.18 ± 3.17
	30	7.8403 ± 0.00007	2917.42 ± 79.56	2157.46 ± 2.34
M-3	40	8.0117 ± 0.00010	2289.79 ± 38.95	2199.01 ± 3.03
	50	8.1929 ± 0.00010	1643.59 ± 20.25	2238.27 ± 3.25
	30	7.3220 ± 0.00010	1839.93 ± 31.93	2003.86 ± 3.32
M-4	40	7.4603 ± 0.00104	1418.65 ± 19.13	2032.82 ± 28.98
	50	7.5207 ± 0.00104	1111.80 ± 9.41	2038.54 ± 28.35

GPU = gas permeation unit = 10^−6^ cc (STP)/cm^2^/s/cm Hg.

**Table 7:** Analysis of variance for total permeation flux and selectivity vs. membrane type and temperature.

Source of Variation	Degree of Freedom (df)	Sum of Squares (SS)	Mean Squares (MS)	F	*p*-Value	F_crit_
** Total Permeation Flux**						
Type of Membrane (M)	4	0.000245	6.13 × 10^−5^	366.7553	5.3 × 10^−25^	2.689628
Temperature (T)	2	5.5 × 10^−5^	2.75 × 10^−5^	164.5891	6.72 × 10^−17^	3.31583
Interaction (M*T)	8	4.14 × 10^−6^	5.18 × 10^−7^	3.098072	0.011378	2.266163
Error	30	5.01 × 10^−6^	1.67 × 10^−7^			
** Selectivity**						
Type of Membrane (M)	4	4,795,879	1,198,970	1080.586	5.99 × 10^−32^	2.689628
Temperature (T)	2	5,714,707	2,857,354	2575.224	2.76 × 10^−34^	3.31583
Interaction (M*T)	8	1,242,278	155,284.7	139.9522	1.34 × 10^−21^	2.266163
Error	30	33,286.67	1109.556			

**Table 8:** Arrhenius activation parameter for permeation, diffusion, and heat of sorption in water–bioethanol mixture.

Parameters (kJ mol^−1^)	M	M-1	M-2	M-3	M-4
E_P_	1.75	1.95	1.33	1.79	1.09
E_pw_	1.58	1.67	0.93	1.50	0.70
E_PE_	9.94	19.61	27.71	24.78	21.19
E_D_	1.59	1.69	0.95	1.51	0.72
E_Dw_	1.58	1.67	0.93	1.50	0.70
E_DE_	9.94	19.61	27.71	24.78	21.19
∆H_S_	0.16	0.26	0.37	0.28	0.37

## Data Availability

The study did not report any data.

## Supplementary Materials

The following supporting information can be downloaded at: https://www.mdpi.com/article/10.3390/polym14235114/s1.

## Nomenclature

*M_w_*	Molecular weight
*A*	Effective membrane area (m^2^)
*D_S_*	Degree of swelling (%)
*D_o_*	Pre-exponential factor for diffusion
E_D_	Activation energy for diffusion (kJ/mol)
E_Dw_	Activation energy for diffusion of water (kJ/mol)
E_DET_	Activation energy for diffusion of bioethanol (kJ/mol)
E_p_	Activation energy for permeation (kJ/mol)
E_pw_	Activation energy for permeation of water (kJ/mol)
E_pET_	Activation energy for permeation of bioethanol (kJ/mol)
E_x_	Activation energy for permeation or diffusion (kJ/mol)
E_T_	Bioethanol
ΔH_s_	Heat of sorption (kJ/mol)
*J*	Total flux (kg/m^2^h)
*J_o_*	Pre-exponential factor for permeation
*PSI*	Pervaporation separation index
*P and F*	Mass percent of permeate and feed
*R*	Gas constant
*t*	Permeation time (h)
*T*	Temperature (K)
*W*	Mass of permeate (kg)
*W_s_ and W_d_*	Mass of the swollen and dry membranes
**Greek letters**	
δ	Membrane thickness (50 μm)
α_sep_	Separation factor
