# Supplementary material for: Novel Polyelectrolyte Complex Membranes Containing Carboxymethyl Cellulose–Gelatin for Pervaporation Dehydration of Azeotropic Bioethanol for Biofuel
Source: Polymers (Basel). 2022 Nov 24;14(23):5114. doi: 10.3390/polym14235114 (PMC9735832; doi:10.3390/polym14235114)
Supplement: Supplementary file 1 [file polymers-14-05114-s001.zip › polymers-1900352-supplementary.pdf]

polymers

MDPI

Supplementary Materials

Novel Polyelectrolyte Complex Membranes Containing Carboxymethyl Cellulose–Gelatin for Pervaporation Dehydration of Azeotropic Bioethanol for Biofuel

Prakash B. Kalahal [1], Ashok M. Sajjan [1,2,*], T. M. Yunus Khan [3], Ali A. Rajhi [3], Sharanappa Achappa [4], Nagaraj R. Banapurmath [2], Ashwini M [5], Alaauldeen A. Duhduh [6]

[1] Department of Chemistry, KLE Technological University, Hubballi 580031, India
[2] Center for Material Science, KLE Technological University, Hubballi 580031, India[3]Department of Mechanical Engineering, College of Engineering, King Khalid University, Abha 61421, Saudi Arabia
[4] Department of Biotechnology, KLE Technological University, Hubballi 580031, India
[5] AICRP on EAAI (Bioconversion Technology) MARS, University of Agricultural Sciences, Dharwad 580005, India
[6] Department of Mechanical Engineering Technology, CAIT, Jazan University, Prince Mohammed Street, P.O. Box 114, Jazan 45142, Saudi Arabia
* Correspondence: am_sajjan@kletech.ac.in; Tel.: +91-944-880-1139; Fax: +91-836-2374985

Membrane characterization

The structural interaction between NaCMC and Ge was studied by Spectrum two with Diamond ATR Fourier transforminfrared (FTIR) spectroscopy (PerkinElmer Singapore Pvt. Ltd., 28, Ayer Rajah Crescent, no. 08-01, Singapore 139959). FTIR analysis was recorded in the range of 500 to 4000 cm^{-1}. The solid-state morphology of the developed membranes was identified using a wide-angle X-ray diffractometer (Rigaku Smart Lab SE, Tokoyo, Japan). The X-ray source was Ni-filtered Cu Kα radiation (30 mA) and 40 kV of cathode current operation was applied. Membrane samples were scanned in the range 5° to 60° for the angle 2θ at the rate of 8°/min. The thermal analysis of the fabricated membranes was assessed by thermogravimetry analysis (TGA) (DSC Q 20, TA Instruments, Waters LLC, USA). 9-10 mg of sample was heated at the rate of 10 ◦C/min under a nitrogen atmosphere. The surface morphology of the fabricated membranes was analyzed using a scanning electron microscope (SEM) analysis named JEOL-JSM-IT500, (Tokyo, Japan). All the membranes were dried properly prior to the analysis and were sufficiently covered with a sputtered gold layer of 400 Å. The mechanical strength and elastic nature of the developed membranes were analysed using the Universal Testing Machine (DAK system Inc., Maharastra, India). Contact angle instrument (Kyowa Contact Angle Meter Model DMS-401, Japan), was used to evaluate the changes in the membrane hydrophilicity. In order to determine the contact angle, Image analysis software (FAMS software) was used.
